# SEOM clinical guidelines for diagnosis and treatment of metastatic colorectal cancer (2018)

**DOI:** 10.1007/s12094-018-02002-w

**Published:** 2018-12-18

**Authors:** M. A. Gómez-España, J. Gallego, E. González-Flores, J. Maurel, D. Páez, J. Sastre, J. Aparicio, M. Benavides, J. Feliu, R. Vera

**Affiliations:** 1 Servicio de Oncología Médica, H. Universitario Reina Sofía, IMIBIC, CIBERONC, Av. Menéndez Pidal, s/n, 14004 Córdoba, Spain; 2 Servicio de Oncología Médica, Hospital General Universitario, Elche, Spain; 3 Servicio de Oncología Médica, H. U. Virgen de las Nieves, Granada, Spain; 4 Servicio de Oncología Médica, Hospital Clinic, Barcelona, Spain; 5 Servicio de Oncología Médica, Hospital de la Santa Creu i Sant Pau, Barcelona, Spain; 6 Servicio de Oncología Médica, Hospital Clínico San Carlos, IdISSC, CIBERONC, Madrid, Spain; 7 Servicio de Oncología Médica, Hospital Universitari i Politècnic La Fe, Valencia, Spain; 8 Servicio de Oncología Médica, H.U. Regional y Virgen de la Victoria, Málaga, Spain; 9 Servicio de Oncología Médica, H. U. La Paz, UAM, CIBERONC, Madrid, Spain; 10 Servicio de Oncología Médica, Complejo Hospitalario de Navarra, IdiSNA, Pamplona, Spain

**Keywords:** Colorectal cancer, Metastases, Surgery, Chemotherapy, Targeted agents, Ablative treatments, Frail patients

## Abstract

Colorectal cancer (CRC) is the second cause of cancer death in Spain, the objective of this guide published by the Spanish Society of Medical Oncology is to develop a consensus for the diagnosis and management of metastatic disease. The optimal treatment strategy for patients with metastatic CRC should be discussed in a multidisciplinary expert team to select the most appropriate treatment, and integrate systemic treatment and other options such as surgery and ablative techniques depending on the characteristics of the tumour, the patient and the location of the disease and metastases.

## Introduction

In Spain, 41,441 new cases of CRC were estimated for 2015, representing the second most common tumour type in both sexes. It was more frequent in men (24,764) than in women (16,677) [1]. Twenty to twenty-five per cent of patients have metastatic disease at initial diagnosis and nearly 50% of patients will eventually develop metastases, which explains the high mortality rates reported for CRC. In fact, this tumour accounts for 13.7% and 14.3% of all cancer deaths in men and women, respectively, in our country. SEOM gathered ten CRC experts based on their major scientific contributions in the field. The purpose of this paper was to define the current “state of the art” through the use of evidence based medicine. The available medical literature was reviewed according to main topics of disease management, and classified by scientific levels of evidence and grades of clinical recommendation (Table 1) [2]. The resulting text was reviewed, discussed, and approved by all authors.

**Table 1:** Levels of evidence and grades of recommendation

Levels of evidence
I. Evidence from at least one large randomized, controlled trial of good methodological quality (low potential for bias) or meta-analyses of well-conducted randomized trials without heterogeneity
II. Small randomized trials or large randomized trials with a suspicion of bias (lower methodological quality) or meta-analyses of such trials or of trials with demonstrated heterogeneity
III. Prospective cohort studies
IV. Retrospective cohort studies or case–control studies
V. Studies without control group, case reports, experts opinions
Grades of recommendation
a. Strong evidence for efficacy with a substantial clinical benefit, strongly recommended
b. Strong or moderate evidence for efficacy but with a limited clinical benefit, generally recommended
c. Insufficient evidence for efficacy or benefit does not outweigh the risk or the disadvantages (adverse events, costs,), optional
d. Moderate evidence against efficacy or for adverse outcome, generally not recommended
e. Strong evidence against efficacy or for adverse outcome, never recommended

## Staging

The extent of the disease must be carefully assessed, as well as tumour biology and patient-related factors before starting cancer-specific therapy.

Table 2 shows suggested staging procedures. The recommended staging system is the 8th edition of the American Join Committee on Cancer (AJCC) [3].

**Table 2:** Suggested staging procedures

Clinical examination
Laboratory tests including liver and renal function tests and prognostic markers (white blood cell count, alkaline phosphatase, lactate dehydrogenase (LDH), bilirubin, and albumin)
Carcinoembryonic Antigen (CEA)
Pathological review of a tumour biopsy (histological subtype, tumour grade, microsatellite status, and *KRAS*, *NRAS* and *BRAF* mutational status)
Computed tomography (CT) scan of the chest, abdomen and pelvis. Magnetic resonance imaging (MRI) could be considered in cases of hepatic metastases and locally advanced rectal tumours
Complete colonoscopy to locate the primary tumour, to obtain tissue for histological diagnosis, and to detect potential synchronous colorectal lesions. Virtual colonoscopy or barium enema could be useful in case of tumours that impede the progression of the endoscopic tube
Other tests such as a bone scan or a brain CT scan should be performed only if clinically indicated
Needle biopsy of a patient with known histologic diagnosis is only recommended when it may change the therapeutic strategy
Additional examinations, as clinically needed, are recommended prior to major abdominal or thoracic surgery with potentially curative intent
Abdominal MRI with intravenous contrast may be considered If liver-directed therapy or surgery is contemplated, and for patients with iodine allergy
A fluorodeoxyglucose (FDG)-positron emission tomography (PET–CT) scan should be performed in the case of potentially surgically curable M1 disease

## Biomarkers

In the mCRC setting, biomarkers may be classified as prognostic or predictive biomarkers.

Prognostic biomarkers: Classical clinical and biochemical parameters such as ECOG performance status, WBC count, alkaline phosphatase (AP), lactate dehydrogenase (LDH) and number of metastatic sites have been considered as the main prognostic factors for survival. Recently, a multivariate analysis carried out by the GERCOR group in patients receiving oxaliplatin or irinotecan first-line combinations has shown that ECOG, LDH and number of metastatic sites are the independent most important clinical prognostic factors [4]. A prognostic model classify patients in low risk (0 points, median OS 29.8 months), intermediate risk (1–2 points, median OS 19.5 months) and high-risk (3–4 points, median OS 13.9 months).

In the last years, primary tumour sidedness has emerged as an important prognostic factor in wild-type RAS patients receiving chemotherapy plus targeted therapy [5]. A significant worse prognosis is associated with right-sided tumours in terms of response rate, progression-free survival and overall survival.

Among the biological biomarkers for survival in patients with metastatic CRC, BRAF mutation V600E is the strongest poor prognostic factor [6]. The negative prognostic role of RAS mutations has consistently been observed regardless the treatment regimen and the addition or not of bevacizumab [6].

Finally, the role of the circulating tumour cell count > 3 in 7.5 ml of blood, by the CellSearch system, as prognostic factor, has been demonstrated in many prospective clinical trials and meta-analysis, but only the FDA has approved this technology for clinical practice [7].

Predictive biomarkers: at the moment, no useful predictive biomarkers have been identified for patients receiving first-line oxaliplatin or irinotecan-based combinations or antiangiogenic drugs. In contrast, activating RAS (KRAS/NRAS) mutations have been identified as biomarkers for cancer cell resistance to monoclonal antibodies directed to epidermal growth factor receptor (EGFR) [8]. As a result, the European Medicines Agency (EMA) has restricted the use of these drugs to patients with RAS wild-type tumours. All the studies that have validated the role of RAS mutations as negative predictive factor for anti-EGFR therapy were done with available archived paraffin tumour samples and then, should be kept as the gold-standard in clinical practice as long as the performing lab complies with nationally and internationally qualified quality assurance programs. High sensitivity technologies to test RAS mutations in ctDNA have observed a high concordance with the standard procedures and might be an alternative when no tumour sample is available or for testing secondary resistance [9].

Preliminary data suggest that high-microsatellite instability (MSI-H) or mismatch repair deficient (dMMR) and HER-2 over-expression or amplification, might have a role as predictive factors for therapy with check-point inhibitor antibodies and trastuzumab plus lapatinib, respectively, but at the moment only check-point inhibitors are approved by the FDA, but not for the EMA as immunotherapy for resistant MSI-H/dMMR colorectal cancer patients.

*Recommendations*: In all patients with metastatic colorectal cancer, exon 2, 3 & 4 mutations in RAS and V600E BRAF mutation should be tested. Furthermore, mismatch repair deficiency (IHC or MSI-H) is recommended to find patients potential candidates for immunotherapy rescue.

## Resectable colorectal metastases

Resectable colorectal liver metastases (RLM) are defined as metastatic liver disease in that a R0 resection can be performed, leaving at least a 20–25% of total liver volume as future liver remanent [10].

Preoperative factors found to be independent predictors of poor survival are a primary tumour at stage T4, ≥ 4 liver metastases, the largest liver metastasis ≥ 5 cm in diameter, and a serum CEA level ≥ 5 ng/ml. According to these factors, resectable patients could be divided in high risk patients (3 or more factors) and low risk patients (less of 3 factors) [11].

The optimal sequencing of systemic therapy and resection in RLM remains unclear. Patients with resectable disease may undergo resection first, followed by postoperative adjuvant chemotherapy. Alternatively, perioperative (neoadjuvant plus postoperative) systemic therapy can be used.

A 2012 meta-analysis identified 3 randomized clinical trials comparing surgery alone to surgery plus systemic therapy. The analysis showed a benefit of chemotherapy in PFS (progression free survival) and DFS (disease free survival) but not in OS (overall survival). Another meta-analysis combined data on 1.896 patients and also found that perioperative chemotherapy improved DFS but not OS. Additional recent meta-analyses have also failed to observe an OS benefit with adjuvant chemotherapy [12].

In low risk patients, perioperative treatment may not be necessary. (I,C) In high risk patients, perioperative combination chemotherapy should be administered. (I,B) In high-risk patients, those who had received neoadjuvant chemotherapy had a better overall median survival (38.9 m vs. 28.4 m) than those had not received it, and 5 years OS rate of 39% vs 33% (*p* = 0.028). In low risk patients, this difference in median survival (60.0 m vs. 60.0 m) was not found according to whatever received preoperative chemotherapy. In the case of low risk patients, who have not received perioperative chemotherapy, there is no strong evidence to support the use of adjuvant chemotherapy (II, C) whereas patients with unfavourable criteria may benefit from adjuvant treatment.

The preferred treatment in RLM should be FOLFOX (or alternatively CAPOX) as reported for the EPOC trial [13] (IV, B). EGFR-targeting monoclonal antibodies are not recommended to be used in this setting, based on the data from the New EPOC trial [14]. No data with bevacizumab are available for this specific patient group.

Colorectal metastatic disease sometimes occurs in the lung. Complete resection based on the anatomic location and extent of disease with maintenance of adequate function is required. Ablative techniques may be considered alone or in conjunction with resection for resectable disease. All original sites of disease need to be amenable to ablation or resection**. **Most of the treatment recommendations discussed for metastatic colorectal liver disease also apply to the treatment of colorectal pulmonary metastases. Combined pulmonary and hepatic resections of resectable metastatic disease have been performed in very highly selected cases.

In patients with limited peritoneal carcinomatosis, complete cytoreductive surgery and HIPEC can be considered in centres with experience.

*Recommendations*: Resection is recommended for resectable liver metastases. Criteria of resectability and prognostic factors are necessary for guiding the need to administer systemic perioperative/adjuvant therapy. Surgery for resectable lung metastases may be considered. Complete cytoreductive surgery and HIPEC can be an option if limited peritoneal carcinomatosis is present.

## Potentially resectable metastatic disease

Patients with initially unresectable, organ-confined liver and/or lung metastases should be considered candidates for secondary surgery. Conversion therapy offers the best means of achieving the goal of resectability. Survival times for patients resected after chemotherapy are slightly shorter than for those patients with upfront resectable disease, but far better than when surgery is not possible at all. Response to systemic therapy is a strong prognostic indicator and correlates well con with resection rates [15]. With the increasing efficacy of current regimens, resectability has to be reevaluated every 2 months (to attain the maximal tumour shrinkage) and surgery scheduled as soon as possible (to minimize chemotherapy-induced liver toxicities and perioperative morbidity). CT morphologic criteria are more predictive of pathological tumour response than RECIST in patients treated with bevacizumab [16].

For fit patients, the most active induction regimens should be administered upfront, generally chemotherapy doublets combined with a biologic agent or chemotherapy triplets plus/minus a biologic. Combinations of either oxaliplatin or irinotecan with fluoropyrimidines are considered adequate options with similar efficacy albeit different toxicity profiles. Patient’s molecular profile should be considered for adding targeted therapies. Comparative trials and meta-analyses suggest that anti-EGFR agents may be more effective in terms of tumour shrinkage than bevacizumab for RAS WT patients [17], especially in left-sided primary tumours [18]. Triplet combination of FOLFOXIRI with or without bevacizumab may be considered in selected (RAS- or BRAF-mutant) patients at the expense of an increased toxicity [19].

**Fig. 1:**
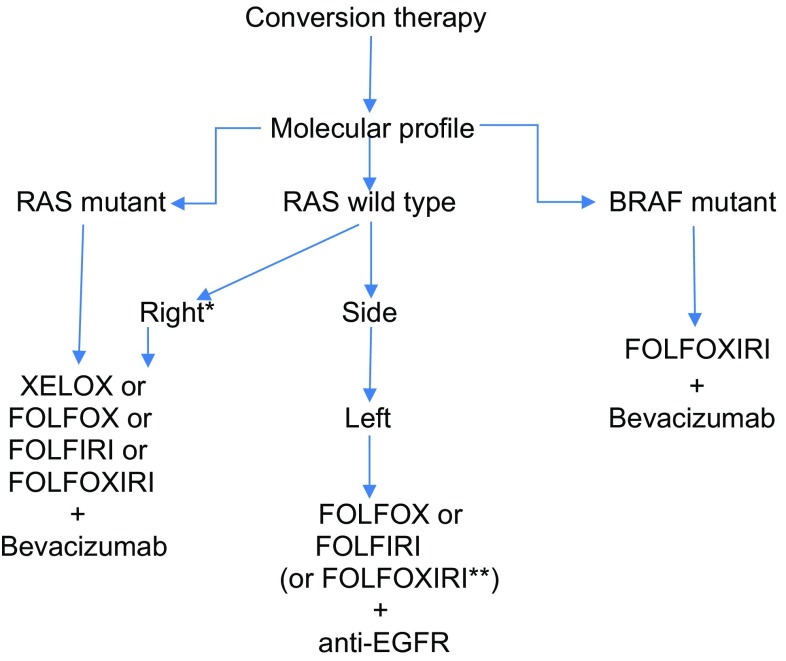
Conversion therapy. * The benefit of adding anti-EGFRs in right-sided RAS wild type metastatic colorectal cancer is controversial. Data from meta-analysis suggest a beneficial effect on response rates but not on survival times. ** Combination of FOLFOXIRI plus panitumumab or cetuximab has not been extensively evaluated

*Recommendations*: Fit patients with borderline resectable metastases should receive intensive induction therapy with chemotherapy doublets and a biologic agent, or chemotherapy triplets with or without biologics (II, A). In RAS WT tumours, anti-EGFRs may be more effective than bevacizumab in terms of tumour shrinkage, especially for left-sided primary tumours (II, B). Figure 1.

## Unresectable disease. First-line treatment

For the purpose of this guideline, treatment with chemotherapy for fit patients with unresectable mCRC at diagnosis or deemed not eligible for radical treatment in the evolution of the disease is considered. In this scenario, main treatment endpoints include prolonging patient survival, relieving symptoms caused by the disease while improving and sustaining quality of life.

Factors considered to influence on first-line treatment decision for unresectable mCRC, once established that all components included in the recommendation should have formal approved indication by national and local regulatory agencies, are patient characteristics and wishes, as well as characteristics of the disease. Unfit patients treatment will be covered anywhere in this guideline, hence we will only concern for patients without conditions which would made them unable to fulfil inclusion criteria for clinical trials that scientifically endorse treatment combinations for first-line treatment of mCRC. Patient personal wishes and circumstances should always be taken into account before establishing any final treatment recommendation. On the other hand, regarding disease characteristics, tumour KRAS, NRAS and BRAF mutational status, as well as tumour sidedness are factors to be considered when selecting first-line treatment for mCRC.

First-line treatment relies on a chemotherapy combination in association with a biologic agent, either bevacizumab or one of the two agents against the epidermal growth factor receptor (EGFR), panitumumab or cetuximab [20, 21]. Chemotherapy combinations include doublets of fluoropyrimidine (5-fluorouracil or capecitabine) and oxaliplatin or irinotecan. Considering all possible treatment combinations, association of capecitabine and irinotecan, as well as association of anti-EGFR antibodies with capecitabine are not recommended. The use of triplet combinations of chemotherapy including 5-fluorouracil, oxaliplatin, and irinotecan (FOLFOXIRI) has demonstrated safety and efficacy in phase III trials, alone or combined with bevacizumab [22] (I, A).

Evidence of tumour RAS (KRAS, NRAS) mutation has demonstrated to be a negative predictive factor for treatment with antibodies against EGFR, cetuximab and panitumumab (I,A). The consideration of tumour BRAF mutation as a negative predictive factor is still nowadays a controversial issue, although analysis done so far suggest lack of benefit of the treatment with panitumumab and cetuximab in these patients [23].

**Fig. 2:**
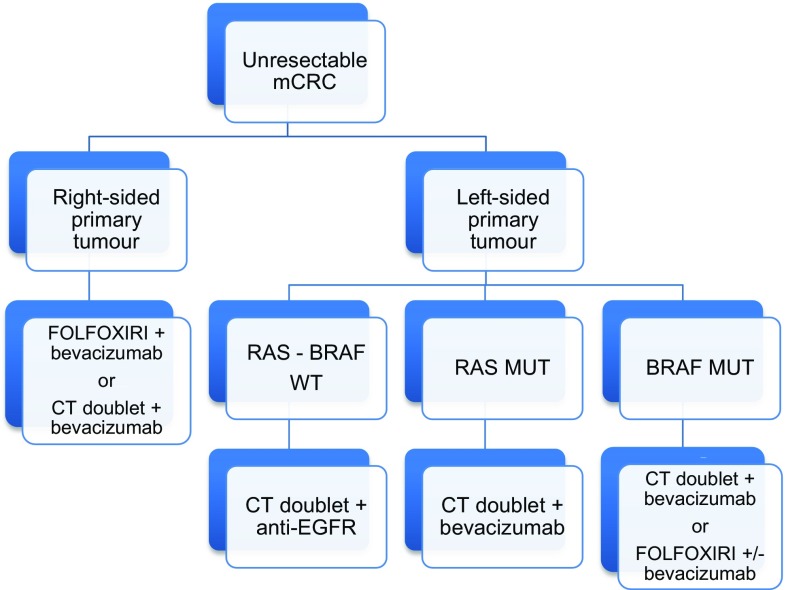
First-line treatment strategy for unresectable metastatic colorectal cancer (mCRC). *WT* Wild type; *CT* chemotherapy, *EGFR* epidermal growth factor receptor, *MUT* mutated

For patients with RAS and BRAF wild type tumours, primary tumour location may influence in treatment selection. Results from the joint analysis of trials of chemotherapy associated to anti-EGFR antibodies demonstrated greater benefit of first-line treatment with these combinations in left-sided tumours; while for right-sided tumours greater benefit in terms of survival is suggested for chemotherapy combined with bevacizumab [5] (I, A) (Fig. 2).

In case of RAS or BRAF mutated tumours, independently of primary tumour location, first-line treatment recommendation is chemotherapy combinations associated with bevacizumab (I, A) (Fig. 2).

Regarding first-line treatment combinations including bevacizumab, intensified chemotherapy with FOLFOXIRI provides greater benefit to patients with right-sided tumours, and this regime has been also suggested as preferred option for BRAF mutated tumours [22] (II, B) (Fig. 2).

Although molecular knowledge of mCRC has introduced new molecular factors to be considered (microsatellite instability, HER2 amplification or mutation, BRAF subgroups, RET fusions, and ALK, ROS, NTR1K rearrangements, among others), whilst generating consensus molecular subgroups of CRC (immune, canonical, metabolic and mesenchymal), none of these have been established in clinical practice for first-line treatment of mCRC thus far.

The recommendation concerning duration of first-line treatment in mCRC, historically considered to be until disease progression, unacceptable toxicity, or patient desire, has been modified in recent years so that nowadays it is subjected to patient personal circumstances, cumulative treatment toxicity, and aggressiveness of the disease. Hence different strategies have emerged, including stop-and-go and intermittent treatment, as well as maintenance treatment consisting of fluoropyrimidines with or without bevacizumab, for patients for whom disease control has been achieved (I,A). The approach to include these treatment options goes through essential individualization and discussion with the patient.

**Fig. 3:**
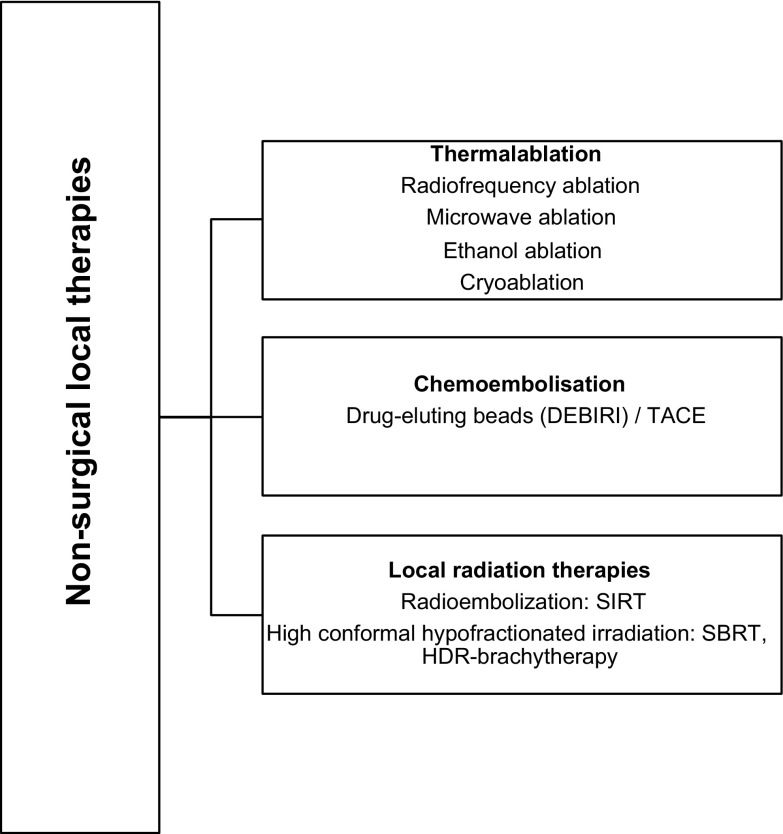
Ablative therapies. *SIRT* selective internal radiation therapy, *SBRT* stereotactic body radiation, *DEBIRI* drug-eluting beads loaded with irinotecan, *TACE* transarterial chemoembolization, *SIRT* selective internal radiation therapy, *HDR* high-dose rate

*Recommendations*: first line treatment for fit patients with unresectable metastatic colorectal cancer should be based upon patient characteristics and wishes, tumour sidedness and RAS y BRAF status.

## Second and successive treatment lines

More than 50% receive second line and more 25% receive third line of treatment. Therapy after first progression will depend on prior treatments, ECOG and adequate organ function, RAS/BRAF status and MSS.

For patients who received oxaliplatin based therapy, FOLFIRI, or irinotecan alone are the preferred options. When the previous treatment was an irinotecan-based combination, the recommended options are FOLFOX or XELOX. With respect to the use of targeted therapies, recommendations are as follows:

Adding bevacizumab or aflibercept to chemotherapy is an option in second-line therapy (I,A) [24, 25]. For patients treated with first-line bevacizumab-containing chemotherapy, the continuation of bevacizumab in conjunction with a second-line chemotherapy improves OS as compared to just switching the chemotherapy regimen alone although the amount of benefit is inferior compared with bevacizumab-naïve patients [26]. Aflibercept added to FOLFIRI in patients previously progressing on a prior oxaliplatin containing regimen is superior to FOLFIRI plus placebo in RR, PFS and OS [25].

Cetuximab and panitumumab appear to have efficacy when used for salvage therapy in all *RAS* WT patients EGFR-naïve chemotherapy-refractory mCRC (II,A) [27, 28]. The reintroduction of EGFR inhibitors in subsequent treatment lines is not recommended for previously exposed patients (I, C).

Regorafenib and TAS 102 give a modest benefit in third line therapy [29, 30] compared with best supportive care, and therefore, its use should be restricted to selected patients (I, B).

Immunotherapy with pembrolizumab [31], nivolumab ± ipilimumab [32, 33] for MSI-H/dMMR (4% of mCRC) could be an option for resistant colorectal cancer patients, but at the moment check-point inhibitors are approved by the FDA, but not by EMA.

*Recommendations*: the second-third line therapies will depend on the prior line, ECOG and organ functions.

## Treatment of frail unresectable mCRC patients

No consensus guidance exists regarding the definition of frailty in patients with cancer, otherwise this term almost always associated with aging. Frailty is associated with dependence, comorbidity, polypharmacy, nutritional and functional status or geriatric syndromes. Treatment results may be biased because acting as a competing cause of death and tolerability in this particularly vulnerable group of patients.

ASCO recently published guidelines for geriatric oncology, recommends a geriatric assessment (GA) for the management of vulnerabilities in older patients (> 65 years) receiving chemotherapy. This GA should include at a minimum, the assessment of function, comorbidity, falls, depression, cognition, and nutrition to obtain estimates of chemotherapy toxicity risk and mortality [34]. However, GA is not a routine in daily practice, but may serve for future research including frail younger patients too.

Evidence-based treatment decisions for unresectable mCRC in this population derives from trials in relatively healthy elderly or different types of frail patients with very few ECOG-2 represented in the trials. To date, integration in a palliative care unit associated or not to specific systemic therapy, is the most important support in these patients.

Both NCCN [35] and ESMO [36] or the adapted for sidedness ESMO-ASIA guidelines [37], recommend some form of therapy in unresectable mCRC patients defined as not appropriate for intensive therapy (NCCN) or unfit (but may be suitable) for ESMO. Fluoropyrimidine monotherapy is the most recommended in patients unable to tolerate aggressive treatment [I,B]. Bevacizumab has been largely study in randomized or not studies and may be added because was safe and increased PFS [38–40].

In wt-*RAS* and left-sided, anti-EGFR (2B NCCN) therapy can be recommended. A Spanish phase II study with first-line single-agent panitumumab in frail elderly patients with wt-KRAS mCRC and poor prognostic factors showed activity and good tolerance and may be an option for patients not candidates for chemotherapy [41].

If treatment is given, dose-attenuated chemotherapy is always recommended and maintenance therapy or chemotherapy holidays may be appropriate. A randomized study of reduced chemotherapy in elderly and/or frail patients with mCRC (38% with both advanced age and frailty) suggested some benefit in PFS, significant increase in the ORR adding oxaliplatin to 5-FU and that the substitution of intravenous FU with capecitabine had no effect on quality of life [42].

Local ablation techniques are generally used for more selected mCRC but may be considered in patients not amenable to surgery (IV, B).

Early palliative care is recommended for patients with metastatic colorectal cancer.

*Recommendations*: for vulnerable or frail patients with unresectable mCRC, best supportive care should made integral part of their management. In unfit (but may be suitable) patients, dose attenuated chemotherapy with or without monoclonal antibodies may be an option according to RAS/BRAF and location.

## Locoregional ablative treatments

Non-surgical approaches for metastases confined to a single organ (most frequently the liver) may offer survival advantages beyond that of systemic therapy alone. If resection is not feasible, image-guide ablation, embolization or stereotactic body radiation therapy (SBRT) are reasonable options. In patients with a limited number of lesions and involved sites, the selection of the best locoregional ablative therapy strategy should be discussed within a multidisciplinary team (Fig. 3).

### Thermal ablation

Although there are many different thermal ablation techniques (microwave ablation, ethanol ablation, cryoablation), the use of radiofrequency ablation (RFA) to treat cancer that has spread to the liver is the best understood. In the phase II CLOCC trial, 119 patients with non-resectable colorectal liver metastases were randomized to systemic treatment or systemic treatment plus RFA. The study met the primary end point on 30-month OS rate > 38%; however, this rate was also achieved in the control arm. RFA plus systemic treatment improved PFS rate at 3 years for combined treatment (27.6% vs. 10.6% for systemic treatment alone; HR, 0.63; 95% CI 0.42–0.95; *p *= 0.025) [43]. For lung metastases from CRC most evidence supports surgery as the most effective treatment option.

### Chemoembolization

Hepatic transarterial chemoembolization (TACE) therapy with drug-eluting beads loaded with irinotecan (DEBIRI) has been used in several prospective studies demonstrating an acceptable toxicity profile. In a randomized phase II trial, median OS was significantly longer for patients treated with DEBIRI than for those treated with FOLFIRI (22 moths vs. 15 months *p *= 0.031) [44]. A recent trial showed that the simultaneous administration of modified FOLFOX (with or without bevacizumab) and DEBIRI significantly increased the objective responses in comparison with the FOLFOX/bevacizumab arm (78% vs 54% at 2 months; *p *= 0.02) [45].

### Radioembolization

Hepatic arterial radioembolization with yttrium-90 bound to resin microspheres plus fluorouracil (FU) was well tolerated and significantly improved time to liver progression compared with FU alone in a small phase III trial in patients with refractory unresectable colorectal liver metastasis [46]. More recently, in a phase III trial (SIRFLOX study), 530 chemotherapy-naïve patients with liver metastases were randomly assigned to receive either modified FOLFOX ± bevacizumab or modified FOLFOX ± bevacizumab plus selective internal radiation therapy using yttrium-90 resin microspheres (SIRT). Although the primary endpoint was not met, SIRT delayed disease progression in the liver (20.5 vs. 12.6 months in the chemotherapy only arm; HR, 0.69; 95% CI 0.55−0.90; *p *= 0.002) [47].

### High conformal hypo fractionated irradiation

In several retrospective and prospective studies, SBRT and high dose rate brachytherapy have been reported to achieve high local control rates. SBRT offers a safe alternative and non-invasive therapeutic option, for the treatment of selected patients not amenable to surgery with limited number of liver or lung metastases.

*Recommendations*:

1. Local ablative techniques such as thermal ablation or high conformal radiation techniques (e.g., SBRT) can be considered for colorectal liver and lung metastases in patients not suitable to surgery. (IV, B).
2. Chemoembolization and radioembolization are options in highly selected patients with predominant hepatic metastases (IV, B and II, B respectively).

## References

[1] Galceran J, Ameijide A, Carulla M, Mateos A, Quirós JR, Roja D (2017). Cancer incidence in Spain, 2015. Clin Trasl. Oncol..

[2] Dykewicz CA (2001). Summary of the guidelines for preventing opportunistic infections among hematopoietic stem cell transplant recipients. Clin Infect Dis.

[3] - Amin, MB, Greene F, Edge S, Byrd DR, Brookland RK, Washington MK, et al. AJCC Cancer Staging Manual (ed. 8th edition). New York: Springer; 2016.

[4] Chibaudel B, Bonnetain F, Tournigand C, Bengrine-Lefevre L, Teixeira L, Artru P (2011). Simplified prognostic model in patients with oxaliplatin-based or irinotecan-based first-line chemotherapy for metastatic colorectal cancer: a GERCOR Study. Oncologist.

[5] Arnold D, Lueza B, Douillard J-Y, Peeters M, Lenz HJ, Venook A (2017). Prognostic and predictive value of primary tumour side in patients with RAS wild-type metastatic colorectal cancer treated with chemotherapy and EGFR directed antibodies in six randomized trials. Ann Oncol.

[6] Modest DP, Ricard I, Heinemann V, Hegewisch-Becker S, Schmiegel W, Porschen R (2016). Outcome according to KRAS-, NRAS- and BRAF- mutations as well as KRAS mutation variants: pooled analysis of five randomized trials in metastatic colorectal cancer by the AIO colorectal cancer study group. Ann Oncol.

[7] Huang X, Gao P, Song Y, Sun J, Chen X, Zhao J (2015). Meta-analysis of the prognostic value of circulating tumor cells detected with the Cell Search System in colorectal cancer. BMC Cancer..

[8] Sorich MJ, Wiese MD, Rowland A, Kichenadasse G, McKinnon RA, Karapetis CS (2015). Extended RAS mutations and anti-EGFR monoclonal antibody survival benefit in metastatic colorectal cancer: a meta-analysis of randomized, controlled trials. Ann Oncol.

[9] Graselli J, Elez E, Caratù G, Matito J, Santos C, Macarulla T (2017). Concordance of blood-and-tumor-based detection of RAS mutations to guide anti-EGFR therapy in metastatic colorectal cancer. Ann Oncol.

[10] Pawlik TM, Choti MA (2007). Surgical therapy for colorectal metastases to the liver. J Gastrointest Surg..

[11] Zhu D, Zhong Y, Wei Y, Ye L, Lin Q, Ren L (2014). Effect of neoadjuvant chemotherapy in patients with resectable colorectal liver metastases. PLoS ONE.

[12] Araujo RLC, Gönen M, Herman P (2015). Chemotherapy for patients with colorectal liver metastases who underwent curative resection improves long-term outcomes: systematic review and meta-analysis. Ann Surg Oncol.

[13] Nordlinger B, Sorbye H, Glimelius B, Poston GJ, Schalag PM, Rougier P (2013). Perioperative FOLFOX4 chemotherapy and surgery versus surgery alone for resectable liver metastases from colorectal cancer (EORTC 40983): long-term results of a randomised, controlled, phase 3 trial. Lancet Oncol..

[14] Primrose J, Falk S, Finch-Jones M, Valle J, O’Reilly D, Siriwardena A (2014). Systemic chemotherapy with or without cetuximab in patients with resectable colorectal liver metastasis: the New EPOC randomised controlled trial. Lancet Oncol..

[15] Adam R, De Gramont A, Figueras J, Guthrie A, Kokudo N, Kunstlinger F (2012). The oncosurgery approach to managing liver metastases from colorectal cancer: a multidisciplinary international consensus. Oncologist..

[16] Chun YS, Vauthey JN, Boonsirikamchai P, Maru DM, Kopetz S, Palavecino M (2009). Association of computed tomography morphologic criteria with pathologic response and survival in patients treated with bevacizumab for colorectal liver metastases. JAMA.

[17] Khattak MA, Martin H, Davidson A, Phillips M (2015). Role of first-line anti-epidermal growth factor receptor therapy compared with anti-vascular endothelial growth factor therapy in advanced colorectal cancer: a meta-analysis of randomized clinical trials. Clin Colorectal Cancer..

[18] Holch JW, Ricard I, Stintzing S, Modest DP, Heinemann V (2017). The relevance of primary tumour location in patients with metastatic colorectal cancer: a meta-analysis of first-line clinical trials. Eur J Cancer.

[19] Loupakis F, Cremolini C, Masi G, Lonardi S, Zagonel V, Salvatore L (2014). Initial therapy with FOLFOXIRI and bevacizumab for metastatic colorectal cancer. N Engl J Med.

[20] Douillard JY, Siena S, Cassidy J, Tabernero J, Burkes R, Barugel M (2014). Final results from PRIME: randomized phase II study of panitumumab with FOLFOX for first-line treatment of metastatic colorectal cancer. Ann Oncol.

[21] Van Cutsem E, Lenz HJ, Köhne CH, Heinemann V, Tejpar S, Melezinek I (2015). Fluoruracil, leucovorin, and irinotecan plus cetuximab treatment and RAS mutation in colorectal cancer. J Clin Oncol.

[22] Cremolini C, Antoniotti C, Lonardi S, Bergamo F, Cortesi E, Tomasello G, et al. Primary Tumor Sidedness and Benefit from FOLFOXIRI plus Bevacizumab as Initial Therapy for Metastatic Colorectal Cancer. Retrospective Analysis of the TRIBE Trial by GONO. Ann Oncol. 2018; April 20. 10.1093/annonc/mdy140. Epub ahead of print. 10.1093/annonc/mdy140 29873679

[23] Sanz-García E, Argiles G, Elez E, Tabernero J (2017). BRAF mutant colorectal cancer: prognosis, treatment and new perspectives. Ann Oncol.

[24] Giantonio BJ, Catalano PJ, Meropol NJ, O’Dwyer PJ, Mitchell EP, Alberts SR (2007). Bevacizumab in combination with oxaliplatin, fluorouracil, and leucovorin (FOLFOX4) for previously treated metastatic colorectal cancer: results from the Eastern Cooperative Oncology Group Study E3200. J Clin Oncol.

[25] Van Cutsem E, Tabernero J, Lakomy R, Prenen H, Prausová J, Maraculla T (2012). Addition of Aflibercept to Fluorouracil, Leucovorin, and Irinotecan Improves Survival in a Phase III Randomized Trial in Patients With Metastatic Colorectal Cancer Previously Treated With an Oxaliplatin-Based Regimen. J Clin Oncol.

[26] Bennouna J, Sastre J, Arnold D, Osterlund P, Greil R, Van Cutsem E (2013). Continuation of bevacizumab after first progression in metastatic colorectal cancer (ML18147): a randomised phase 3 trial. Lancet Oncol..

[27] Sobrero AF, Maurel J, Fehrenbacher L, Scheithauer W, Abubakr YA, Lutz MP (2008). EPIC: phase III trial of cetuximab plus irinotecan after fluoropyrimidine and oxaliplatin failure in patients with metastatic colorectal cancer. J Clin Oncol.

[28] Peeters M, Price TJ, Cervantes A, Sobrero AF, Ducreux M, Hotko Y (2010). Randomized phase III study of panitumumab with fluorouracil, leucovorin, and irinotecan (FOLFIRI) compared with FOLFIRI alone as second-line treatment in patients with metastatic colorectal cancer. J Clin Oncol.

[29] Grothey A, Van Cutsem E, Sobrero A, Siena S, Falcone A, Ychou M (2013). Regorafenib monotherapy for previously treated metastatic colorectal cancer (CORRECT): an international, multicentre, randomized, placebo-controlled, phase 3 trial. Lancet.

[30] Mayer RJ, Van Cutsem E, Falcone A, Yoshino T, García-Carbonero R, Mizunuma N (2015). Randomized trial of TAS-102 for refractory metastatic colorectal cancer. N Engl J Med.

[31] Le DT, Uram JN, Wang H, Bartlett BR, Kemberling H, Eyring AD (2015). PD-1 Blockade in Tumors with Mismatch-Repair Deficiency. N Engl J Med.

[32] Overman MJ, McDermott R, Leach JL, Lonardi S, Lenz HJ, Morse MA (2017). Nivolumab in patients with metastatic DNA mismatch repair-deficient or microsatellite instability-high colorectal cancer (CheckMate 142): an open-label, multicentre, phase 2 study. Lancet.

[33] Overman MJ, Lonardi S, Wong KYM, Lenz HJ, Gelsomino F, Aglietta M (2018). Durable clinical benefit with nivolumab plus ipilimumab in DNA mismatch repair-deficient/microsatellite instability-high metastatic colorectal cancer. J Clin Oncol.

[34] Mohile SG, Dale W, Somerfield MR, Schonberg MA, Boyd CM, Burhenn PS (2018). Practical Assessment and Management of Vulnerabilities in Older Patients Receiving Chemotherapy: ASCO Guideline for Geriatric Oncology. J Clin Oncol.

[35] - NCCN Clinical Practice Guidelines in Oncology (NCCN Guidelines^®^). NCCN.org Colon Cancer Version 2.2018, March 14, 2018.

[36] Van Cutsem E, Cervantes A, Adam R, Sobrero A, Van Krieken JH, Aderka D (2016). ESMO consensus guidelines for the management of patients with metastatic colorectal cancer. Ann Oncol.

[37] Yoshino T, Arnold D, Taniguchi H, Pentherousdakis G, Yamazaki K, Xu RH (2018). Pan-Asian adapted ESMO consensus guidelines for the management of patients with metastatic colorectal cancer: a JSMO–ESMO initiative endorsed by CSCO. KACO, MOS, SSO and TOS Annals of Oncology..

[38] Aparicio T, Bouché O, Taieb J, Maillard E, Kirscher S, Etienne PL (2018). Bevacizumab chemotherapy versus chemotherapy alone in elderly patients with untreated metastatic colorectal cancer: a randomized phase II trial PRODIGE 20 study results. Ann Oncol.

[39] Cunningham D, Lang I, Marcuello E, Lorusso V, Ocyirk J, Shin DB (2013). Bevacizumab plus capecitabine versus capecitabine alone in elderly patients with previously untreated metastatic colorectal cancer (AVEX): an open-label, randomised phase 3 trial. Lancet Oncol..

[40] Price TJ, Zannino D, Wilson K, Simes RJ, Cassidy J, Van Hazel GA (2012). Bevacizumab is equally effective and no more toxic in elderly patients with advanced colorectal cancer: a sub- group analysis from the AGITG MAX trial: an international randomised controlled trial of capecitabine, bevacizumab and mitomycin C. Ann Oncol.

[41] Sastre J, Massutí B, Pulido G, Guillén-Ponce C, Benavides M, Manzano JL (2015). First-line single-agent panitumumab in frail elderly patients with wild-type KRAS metastatic colorectal cancer and poor prognostic factors: a phase II study of the Spanish Cooperative Group for the Treatment of Digestive Tumours. Eur J Cancer.

[42] Seymour MT, Thompson LC, Wasan HS, Middleton G, Brewster AE, Shepherd SF (2011). Chemotherapy options in elderly and frail patients with metastatic colorectal cancer (MRC FOCUS2): an open-label, randomised factorial trial. Lancet.

[43] Ruers T, Punt C, Van Coevorden F, Pierie JP, Borel-Rinkes I, Ledermann JA (2012). Radiofrequency ablation combined with systemic treatment versus systemic treatment alone in patients with non-resectable colorectal liver metastases: a randomized EORTC Intergroup phase II study (EORTC 40004). Ann Oncol.

[44] Fiorentini G, Aliberti C, Tilli M, Mulazzani L, Graciano F, Giordani P (2012). Intra-arterial infusion of irinotecan-loaded drug-eluting beads (DEBIRI) versus intravenous therapy (FOLFIRI) for hepatic metastases from colorectal cancer: final results of a phase III study. Anticancer Res.

[45] Martin RC, Scoggins CR, Schreeder M, Rilling WS, Laing CJ, Tatum CM (2015). Randomized controlled trial of irinotecan drug-eluting beads with simultaneous FOLFOX and bevacizumab for patients with unresectable colorectal liver-limited metastasis. Cancer.

[46] Hendlisz A, Van den Eynde M, Peeters M, Maleux G, Lambert B, Vannoote J (2010). Phase III trial comparing protracted intravenous fluorouracil infusion alone or with yttrium-90 resin microspheres radioembolization for liver-limited metastatic colorectal cancer refractory to standard chemotherapy. J Clin Oncol.

[47] Van Hazel GA, Heinemann V, Sharma NK, Findlay MP, Ricke J, Peeters M (2016). SIRFLOX: randomized phase III trial comparing first-line mFOLFOX6 (plus or minus bevacizumab) versus mFOLFOX6 (plus or minus bevacizumab) plus selective internal radiation therapy in patients with metastatic colorectal cancer. J Clin Oncol.

